# Association of Static Posturography With Severity of White Matter Hyperintensities

**DOI:** 10.3389/fneur.2021.579281

**Published:** 2021-02-11

**Authors:** Bin Liu, Guifeng Zhao, Ling Jin, Jingping Shi

**Affiliations:** ^1^Department of Geriatrics, The Affiliated Brain Hospital of Nanjing Medical University, Nanjing Brain Hospital, Nanjing, China; ^2^Department Key Laboratory of Research and Application of Animal Models for Environmental and Metabolic Diseases, Sheng Jing Hospital of China Medical University, Shenyang, China; ^3^Department of Neurology, The Affiliated Brain Hospital of Nanjing Medical University, Nanjing Brain Hospital, Nanjing, China

**Keywords:** posturography, balance, white matter hyperintensities, cognition, stroke

## Abstract

**Background:** Impaired gait and balance are associated with severity of leukoaraiosis. Evaluation of balance is based on neurological examination using Romberg's test with bipedal standing, assessment scale, and posturographic parameters. The goal of this study was to determine the relationship between static equilibrium and grades of white matter hyperintensities (WMHs) using static posturography as a quantitative technical method.

**Method:** One hundred and eighteen (118) patients with lacunar infarct were recruited and assessed on MRI with Fazekas's grading scale into four groups. On admission, age, gender, height, weight, Berg Balance Scale (BBS), mini-mental state examination (MMSE), and static posturography parameters were recorded, and their correlations with WMHs were determined.

**Results:** Age was significantly and positively correlated with severity of WMHs (*r* = 0.39, *p* < 0.05). WMH score was negatively correlated with BBS score (*r* = −0.65, *p* < 0.05) and MMSE score (*r* = −0.79, *p* < 0.05). There was a significant positive correlation between track length anteroposterior (AP, with eyes closed) and severity of WMHs (*r* = 0.70, *p* < 0.05). Partial correlation analysis and multiple logistic regression analysis indicated that track length AP with eyes closed, was a predictor for the severity of WMHs (*p*< 0.05).

**Conclusion:** The severity of WHMs is associated with age, cognitive decline, and impairment in balance. Posturography parameter in track length in AP direction with eyes closed in relation to cognition and balance, may be a potential marker for disease progression in WMHs.

## Introduction

Changes in cerebral white matter, also called white matter hyperintensities (WMHs), which are seen in one-third of the ischemic stroke population, are risk factors for acute stroke (1). Although the symptom of WHMs is not apparent, and is not related to hemiplegia and paresthesia, WMHs contribute significantly to cognitive decline and balance impairment as the disease progresses (2). In recent years, the relationship amongst white matter lesions and cognition, physical function and falls have also been investigated, and is thought to result from chronic brain ischemia/small vessel disease (3–6). Therefore, it is necessary to evolve a useful method for assessment of neurofunction deficit in WMHs, particularly in cognition and balance.

A growing body of evidence suggests that postural instability and falls are common and devastating problems in older adults (7). Some clinical tests have been developed to determine the presence and severity of postural instability. The most widely used and recognized balance measure is the Berg Balance Scale (BBS) which predicts whether patients can walk without the need for physical assistance, or at speeds suitable for the community (8). Physiatrists have advocated the BBS as a useful tool for evaluation of the effectiveness of physical therapeutic interventions and for quantitative descriptions of function in clinical practice and research. However, the subjective and variable nature of this balance scoring system make it unreliable.

Static posturography could be used for accurate and objective measurement and analysis of subtle changes in reflexes in maintaining balance, with specialized techniques (9). It is a useful tool for unraveling details of the underlying pathophysiology in patients with balance disorders. A study by Panyakaew has shown the differentiating characteristics of posturography in ambulatory atypical parkinsonian disorders (10). In previous research, posturography was found to be associated with asymptomatic cerebrovascular damage (11). However, the relationship between static posturography in WMHs and the traditional balance scoring system is unclear. The correlation between static posturography parameters and different grades of WMHs has not been ascertained. Therefore, the present study was carried out to objectively test whether quantitative posturographic analysis could be used as a potential marker of disease progression in patients with WMHs.

## Methods

### Study Population

A total of 118 patients admitted and treated in the Department of Geriatric Neurology were prospectively recruited from the Affiliated Brain Hospital to Nanjing Medical University between January 1, 2019 and December 31, 2019. The criteria used were: age above 18 years; patients diagnosed with lacunar stroke according to the TOAST criteria through magnetic resonance imaging (MRI), and patients who were referred with chief complaints about cognitive deficit and imbalance not directly related to the presence lacunar stroke. This study excluded patients in the following categories: (1) patients with contraindications to MRI; (2) patients with WMHs from other identified causes; (3) patients with hemorrhage, severe stroke, Alzheimer's disease, Parkinson's syndrome, paralysis, and aphasia; (4) patients with evidence of a cardiac embolic source or cerebral large vessel disease (at least one internal carotid artery stenosis of 50% on MR angiography or CT angiography); (5) patients with orthopedic diseases, peripheral neuropathy and proprioceptive loss according to physical examination for the nervous system, MRI for spinal cord, and electromyography examination; (6) patients with benign positional vertigo, Meniere's Disease and vestibular neuritis and other peripheral vertigo disease according to positioning test and the video head impulse test (Type 1085, GN/otometrics). The video impulse test was performed as follows: patients sat on chairs, wearing the head pulse test system, looking at the target 120 cm in front. The clinician performed transient, passive and rapid head pulse motion on the plane of the horizontal semicircular canal (left and right), about 15° in about 100 ms. Sensors of the test system detected eye and head movements. Gain was calculated by comparing the areas under the head movement and eye movement curves (12). The patient's gain for the horizontal canal was between 0.8 and 1.2, which was in the normal range. Written informed consent was obtained from each participant, and approval for the study was received from the Ethical Committee of the Affiliated Brain Hospital to Nanjing Medical University. Based on visual rating of the Fazekas's grading scale including periventricular and deep white matter from Magnetic Resonance Imaging (MRI) (13), the patients were divided into four groups: grade 0 for those without WMHs (total score of 0, *n* = 28); grade 1 for patients with mild WMHs (focal or punctate lesions, *n* = 29); grade 2 for patients with moderate WMHs (early confluent lesions, *n* = 29), and grade 3 for those with severe WMHs (confluent lesions, *n* = 32).

### Baseline Characteristics

Clinical data were collected from all patients, with respect to age, gender, height, weight, Berg Balance Scale (BBS), and mini-mental state examination (MMSE). The Berg Balance Scale (BBS) is a 14-item scale which quantitatively assesses balance and risk of falls. The items are scored from 0 to 4 for a total score of 56. High scores in this test indicate excellent balance. Mini-mental state examination (MMSE) is a 10-item screening tool for evaluation of cognitive function. It is 30-point scale for assessing orientation, memory, attention, calculation, and language.

### Assessment of Postural Instability

**Figure 1:**
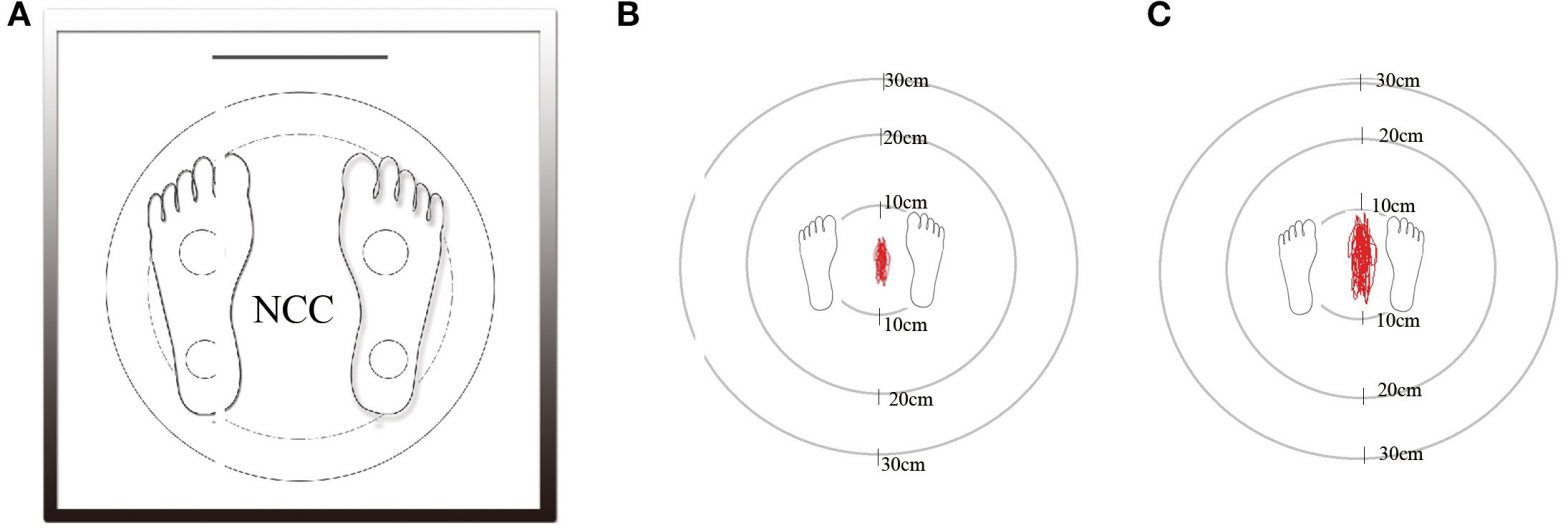
The static posturography platform (Balance-B, NCC, China) is a computerized coordination and balance analysis system with the help of four piezoelectric power sensors to measure the position and movement of the projection of the center of gravity **(A)**. Marked area in a patient of grade 0 without WMHs as control group **(B)**. Marked area in a patient of grade 3 with severe WMHs **(C)**.

Postural instability was measured using a digital platform consisting of two-foot plates with four in-built vertical force transducers for determination of instantaneous fluctuations in the center of pressure (Figure 1A; Balance-B, NCC, China). Each individual was placed on the posturography platform to maintain an upright standing position, with arms at their sides, in a comfortable and quiet environment (14). With eyes open (EO), subjects were asked to look straight at a fixed point on the wall 1 m away for 30 s. In order to remove visual input, all subjects were asked to repeat the same procedure with eyes closed (EC). Changes in positions and the Centre of Pressure (CoP) trajectories were calculated using a software (Figures 1B,C). The CoP was computed from the vertical forces. The CoP is the point location of the ground reaction force vector and reflects the sway of the body and forces used to maintain the center of gravity within the support base. Six dependent variables were calculated: (1) total track length (cm), (2) sway area (cm^2^), (3) track length mediolateral (ML) (cm), (4) track length anteroposterior (AP) (cm), (5) track length per unit area (cm/cm^2^), and (6) average velocity (cm/sec).

### Statistical Analysis

All statistical analyses were performed using the SPSS (Windows, version 16.0 SPSS Inc). Categorical variables for gender are expressed as counts (percentage). Numerical variables are presented as mean ± standard deviation. Chi-square statistics for gender and bivariate correlation analysis (Spearman correlation coefficient) for numeric variables were conducted to determine their correlations with different grades of WMHs. The associations between track length AP in eye closed and age, BBS and MMSE were determined using bivariate correlation analysis (Pearson correlation coefficient). To further evaluate the association between static posturography and WHMs grades, partial correlation analysis and logistic regression analyses were carried out. The *p*-values were two-sided. Differences were assumed as statistically significant at *p* < 0.05.

## Results

### Correlation Between Clinical Characteristics and WMHs Grades

The clinical features which were correlated with WMHs grades are summarized in Table 1. Overall, according to the Fazekas's grading scale, 118 patients comprising 28 cases in grade 0, 29 cases in grade 1, 29 cases in grade 2, and 32 cases in grade 3, completed investigation. Chi-square statistics for gender and bivariate correlation analysis were used to assess their relations with different grades of WMHs. It was found that age had a significant positive correlation with WMHs grades (*r* = 0.39, *p* < 0.05). The BBS scores indicated that deficits in balance significantly decreased in different grades (*r* = −0.65, *p* < 0.05). In addition, there was a negative correlation between MMSE score and WMHs grade (*r* = −0.79, *p* < 0.05). There were no significant differences in gender, height, and weight amongst patients in the different grades.

**Table 1:** Demographic data and clinical features correlated with WMHs grades.

**Variable**	**Grade 0**	**Grade 1**	**Grade 2**	**Grade 3**	**r/χ^2^**	***P***
Gender (%, female)	14 (50)	18 (62)	16 (55)	12 (37.5)	3.97	0.26
Age (years)	63.11 ± 9.50	70.90 ± 10.20	72.00 ± 10.41	74.56 ± 7.67	0.39	0.00^**^
Height (cm)	163.8 ± 6.8	162.3 ± 6.6	164.1 ± 7.8	165.5 ± 7.8	0.11	0.25
Weight (kg)	65.1 ± 7.7	63.7 ± 5.7	64.5 ± 7.1	66.6 ± 7.0	0.09	0.35
BBS	53.43 ± 2.20	53.79 ± 2.37	48.41 ± 4.17	44.81 ± 5.42	−0.79	0.00^**^
MMSE	27.68 ± 1.57	24.59 ± 1.80	19.93 ± 6.00	19.52 ± 3.89	−0.65	0.00^**^

*Categorical variables for gender are expressed as counts (percentage). Numerical variables are presented as mean ± standard deviation. r: spearman coefficient. BBS, Berg Balance Scale BBS; MMSE = mini-mental state examination. The patients were divided into four groups according to the Fazekas's grading scale standard*.

** *p < 0.05 (correlation of variables to WMHs grades)*.

### Static Posturography Parameters in WMHs

To investigate correlation between static posturography and severity in WMHs patients, correlation analysis was used (Table 2). There was a significant positive correlation between track length AP in eye closed, and severity of WMHs (*r* = 0.70, *p* < 0.05). As shown in Table 3, track length AP was significantly correlated with age (*r* = 0.27, *p* < 0.05); BBS (*r* = −0.41, *p* < 0.05), and MMSE (*r* = −0.54, *p* < 0.05). In order to evaluate the potential influences of age, BBS, and MMSE, correlation analysis between track length AP and WMHs grades was repeated using partial correlation analysis, after adjusting for age, BBS, and MMSE. The correlation between track length AP and WMHs grades remained significant (*r* = 0.33, *p* < 0.05) in Table 4.

**Table 2:** Correlation of static posturography parameters with WMHs grades.

**Variable**	**Grade 0**	**Grade 1**	**Grade 2**	**Grade 3**	**r**	***p***
**Eyes opened**						
Total track length (cm)	3.54 ± 1.09	4.94 ± 1.93	4.83 ± 1.66	4.47 ± 1.82	0.18	0.06
Sway area (cm^2^)	1.69 ± 1.39	2.19 ± 1.37	2.56 ± 1.88	2.53 ± 2.28	0.18	0.06
Track length ML (cm)	1.34 ± 0.54	1.76 ± 0.83	1.97 ± 1.13	1.87 ± 1.27	0.17	0.07
Track length AP (cm)	3.04 ± 1.14	4.30 ± 1.66	3.88 ± 1.21	3.71 ± 1.43	0.16	0.09
Track length per unit area (cm/cm^2^)	4.55 ± 0.72	4.36 ± 1.08	4.76 ± 0.82	5.16 ± 1.73	0.17	0.07
Average velocity (cm/sec)	0.14 ± 0.05	0.19 ± 0.07	0.17 ± 0.05	0.17 ± 0.07	0.11	0.25
**Eyes closed**						
Total track length (cm)	5.29 ± 1.83	6.74 ± 3.29	6.86 ± 3.14	6.82 ± 3.97	0.12	0.19
Sway area (cm^2^)	2.68 ± 2.12	3.71 ± 2.96	3.37 ± 1.76	4.56 ± 8.73	0.097	0.31
Track length ML (cm)	1.64 ± 0.69	1.89 ± 0. 69	2.39 ± 2.14	2.67 ± 2.78	0.13	0.18
Track length AP (cm)	3.49 ± 0.76	5.35 ± 1.25	6.66 ± 1.84	6.77 ± 1.86	0.70	0.00^**^
Track length per unit area (cm/cm^2^)	5.55 ± 2.40	6.61 ± 2.56	6.53 ± 1.98	7.12 ± 5.60	−0.02	0.10
Average velocity(cm/s)	0.18 ± 0.06	0.27 ± 0.17	0.25 ± 0.10	0.24 ± 0.14	0.16	0.09

*Six dependent variables of static posturography were calculated: (1) total track length (cm); (2) sway area (cm^2^); (3) track length mediolateral (ML) (cm); (4) track length anteroposterior (AP) (cm); (5) track length per unit area (cm/cm^2^); and (6) average velocity (cm/sec)*.

*r: spearman coefficient*;

** *p < 0.05 (correlation of variables to WMHs grades)*.

**Table 3:** Correlations amongst track length AP (cm, eyes closed) and age, BBS, and MMSE.

	**Track length AP (cm, eye closed)**	
**Variables**	**r**	***p***
Age	0.27	0.00^*^
BBS	−0.41	0.00^*^
MMSE	−0.54	0.00^*^

*R: Pearson coefficient*;

* *p < 0.05: level of significance; mini-mental state examination (MMSE)*.

**Table 4:** Correlation between Track length AP (cm, eye closed) and WMHs grades adjustments by age, BBS and MMSE.

	**WMHs grades**	
**Variables**	**r**	***P***
Track length AP (cm; eye closed)	0.33	0.00^**^

*r: spearman coefficient*;

** *p < 0.05*.

### Logistic Regression Analyses

Based on the results of correlation analysis, logistic regression analyses were carried out to identify independent indicators of grades in WMHs. As shown in Table 5, age, BBS, MMSE and track length AP in eye closed were significant and independent indicators of grades in WMHs (*p* < 0.05).

**Table 5:** Logistic regression analysis of factors and WMHs grades.

**Factor**	**β**	**OR**	**Wald**	***p***	**95% CI**
Age	0.043	1.04	4.30	0.038	0.002–0.083
BBS	−0.27	0.76	28.11	0.00	−0.38–(−0.18)
MMSE	−0.17	0.85	10.28	0.001	−0.26–(−0.064)
Track length AP (cm; eyes closed)	0.61	1.83	14.01	0.00	0.29–0.92

*CI, confidence interval; OR, odds ratio*.

## Discussion

In this study, the potential clinical use of static posturography in the assessment of severity of WHMs was determined. There was a positive correlation between age and WMHs severity. In different grades of WMHs, there was a significant negative correlation between balance and cognitive function represented by BBS and MMSE, respectively. Posturography parameter in track length in AP with eye closed was related to age, BBS, and MMSE, indicating the importance of evaluating severity of WMHs using partial correlation analysis and logistic regression analyses.

It is known that WMHs are highly prevalent in the elderly, and they increase the risk of acute stroke, Alzheimer's disease, and Parkinson's disease (15–17). These are consistent with the results obtained in the present study. In clinical studies, gait and balance impairment are considered the second most common functional deficit in WMHs, in addition to cognitive impairment. Some studies have shown that changes in white matter are strongly associated with severity of compromise in gait and motor function (18). Impairments in gait and balance increase the risk of falls, and they are independent predictors of WMHs (19). Increasing evidence from previous studies have revealed the coexistence of abnormality in balance and cognitive decline in older adults with WMHs (20). White matter fiber tracts are frontal subcortical and periventricular brain circuits which control the capacity of neuromuscular interaction referred to as balance and cognition, and maintain multisystem coordination (21). In a recent study, an imbalance in the presence of subjective cognitive complaints without any form of dementia or mobility disability has been defined as motoric cognitive risk (MCR) syndrome (22, 23). Four criteria have been proposed for MCR: (1) presence of subjective cognitive complaints, (2) existence of imbalance, (3) preserved mobility, and (4) absence of dementia. The MCR syndrome is a predictor of dementia, high frequency of falls, and a high risk of disability (24). In this study, correlation analysis and logistic regression analyses indicated that BBS and MMSE are independent predictors for different grades of WMHs. Data from BBS and MMSE indicated balance impairment and cognitive decline in WMHs, respectively, suggesting an increased MCR risk in WMHs. Evaluation of balance and cognition for patients in the early stage of WMHs could be helpful in providing necessary treatments so as to decrease the risk of falls and delay the progression of cognitive impairment. In the partial correlation analysis, the correlation between track length AP and WMH grade is also moderate (*r* = 0.33), after adjusting for age, BBS and MMSE. In the results logistic regression analyses, track length AP with eyes closed was identified as an independent indicator of grades in WMHs. Those results indicated static posturography could be applied as a new accurate and objective assessment method of balance for WMHs severity.

Assessment of locomotion based on the overall coordination of balance involves the vestibular, kinesthetic, visual, and tactile analyzer (25). Therefore, it is necessary to determine balance based on vestibular-ocular reflex and vestibulospinal reflex. The clinically-used balance assessment is a combination of history taking, physical examination, and subjective scoring system, but these approaches are not infallible. Posturography involves the assessment of the combined influence of gravity and small self-initiated corrective movements, and it is undoubtedly a sensitive clinical measure of balance (26, 27). In a study by Sataloff, dynamic computerized posturography was used to detect white matter change-associated balance dysfunction in WMHs. Dynamic techniques seem to have been used more often in studies of white matter lesions (28). However, static posturography, as an assessment method for balance function in WMHs, is also necessary. In Baloh, R. W.'study, static posturography is less affected by age than dynamic posturography (29). That would be helpful to explore the effect of the static posturography parameter as an independent predictor for WMHs. Visual input which plays an important role in maintaining balance could be withdrawn by simple eye closure in static posturography. In this study, significant differences were detected in track length AP when the eyes were closed in different grades of WMHs. Moreover, in partial correlation and logistic regression analyses, with closed eyes, track length AP had a significant positive correlation with WMHs grades. Although vestibulo-ocular reflex and vestibulo-spinal reflex are very tightly correlated with decline in vestibular function for many vestibular disorders, declined balance function in WMHs showed weak relationship with the visual input, it may be related closely to vestibulospinal reflex in WMHs without the visual input. In addition, static posturography is a simple and time-saving detection method. The entire process with high repeatability, takes only 3 min. Furthermore, the index of this detection method is relatively representative. The sway in AP direction indicated more influence in vestibular impairment, when compared to the sway in ML direction which predicted relationship to cerebellum disease. It seems that track length AP with eyes closed is the best parameter in postural instability for patients with WMHs (30). Therefore, static posturography can easily help to identify balance problems for WHMs assessment in clinical routine, especially with the setting marked with eye closed in AP direction.

In this study, it was found that WMHs were associated with age, cognitive decline, and abnormality in balance. Thus, static posturography may be an accurate and objective assessment of the presence of an imbalance in WMHs. Nonetheless, this investigation has some limitations. In the first place, it is a single-hospital study. Therefore, the possibility of case selection bias cannot be ruled out. Thus, a multi-center clinical trial is needed. Secondly, track length AP with eyes closed is a sensitive index but other parameters could be significantly different in WMHs if the number of samples is increased. Besides, there is need for research on the associated pathway in vestibulospinal reflex and cognitive decline. Mild proprioceptive loss and peripheral neuropathy might influence AP track length eyes closed, as study participants included those aged over 80 and with diabetes. In the future, prospective studies should explore combining a number of physiological and anatomical assessments, to include static posturography, functional MRI, and specific tests of vestibular function such as video head impulse test and vestibular evoked myogenic potentials.

## Conclusion

Static posturography may be an accurate and objective assessment method for balance in aged patients with WMHs. It may offer a new target for practical evaluation and further treatment of patients with balance deficits in WMHs.

## Data Availability Statement

The raw data supporting the conclusions of this article will be made available by the authors, without undue reservation.

## Ethics Statement

The studies involving human participants were reviewed and approved by Ethics Committee of Nanjing Brain Hospital. The patients/participants provided their written informed consent to participate in this study.

## Author Contributions

GZ and LJ: Participation in the whole work, drafting of the article, and data analysis. BL and JS: perception and design, operation, and final approval of the version to be published. All authors contributed to the article and approved the submitted version.

## Conflict of Interest

The authors declare that the research was conducted in the absence of any commercial or financial relationships that could be construed as a potential conflict of interest.
